# High energy proton micro-bunches from a laser plasma accelerator

**DOI:** 10.1038/s41598-019-50348-0

**Published:** 2019-09-25

**Authors:** Ashutosh Sharma, Christos Kamperidis

**Affiliations:** ELI-ALPS, ELI-HU Non-Profit Ltd., Dugonics ter 13, H-6720 Szeged, Hungary

**Keywords:** Laser-produced plasmas, Plasma-based accelerators

## Abstract

Recent advances on laser-driven ion accelerators have sparked an increased interest in such energetic particle sources, particularly towards the viability of their usage in a breadth of applications, such as high energy physics and medical applications. Here, we identify a new ion acceleration mechanism and we demonstrate, via particle-in-cell simulations, for the first time the generation of high energy, monochromatic proton micro-bunches while witnessing the acceleration and self-modulation of the accelerated proton beam in a dual-gas target, consisting of mixed ion species. In the proposed ion acceleration mechanism due to the interaction of an ultra-short, ultra-intense (2 PW, 20 fs) laser pulses with near-critical-density partially ionized plasmas (C & H species), we numerically observed high energy monochromatic proton microbunches of high quality (peak proton energy 350 MeV, laser to proton conversion efficiency ~10^−4^ and angular divergence <10 degree), which can be of high relevance for medical applications. We envisage that through this scheme, the range of attained energies and the monochromaticity of the accelerated protons can be increased with existing laser facilities or allow for laser-driven ion acceleration investigations to be pursued at moderate energies in smaller scale laser laboratories, hence reducing the size of the accelerators. The use of mixed-gas targets will enable high repetition rate operation of these accelerators, free of plasma debris and electromagnetic pulse disruptions.

## Introduction

Laser-ion acceleration has been promptly developing field with the growing installation of PW class lasers. The striking characteristics of laser accelerated ion stimulated a eclectic choice of applications in nuclear^1^ and medical physics^2–4^. The prerequisite of a reproducible, high-repetition rate ion beam with fine energy spread and better efficiency is a challenging mission irrespective of extensive efforts^5^, primarily due to target behaviour with the interaction with modern high-repetition rate laser systems. Laser driven ion acceleration mechanism^6–31^ includes the target normal sheath acceleration (TNSA)^6–8^, radiation pressure acceleration (RPA)^9–17^, breakout afterburner (BOA)^18^, collisionless shockwave acceleration (CSA)^19–21^ and magnetic vortex acceleration (MVA)^22–25^.

TNSA^6–8^ is one of the most stable and well-understood mechanism, which necessitates long pulse durations and thin solid targets to approach high cut-off energies. By employing the solid-density planar target geometries^32^, the highest proton beam quality has been observed with TNSA mechanism and highest proton energies experimentally reported with TNSA are 85 MeV^33^. These ion beams have an inherently broad, thermal energy distribution whereas many potential applications of these compact sources require some degree of spectral control. This is motivating to investigate the new approaches to control the ion energy distribution at the source. Utilising foil targets at high repetition rates raises significant challenges with debris, electromagnetic pulse generation (EMP), target insertion accuracy at the laser focus and unwanted secondary radiation such as bremsstrahlung. Thus, the target choice also needs to avoid or suppress build-up of debris on optical elements in the target chamber to facilitate continuous use for extended periods of time.

In contrast to distinctive ion acceleration from laser-thin solid foil interaction, efficient ion acceleration has also been realized in near critical density (NCD) plasmas from high density gas jets^26^ which are measured to have gain of higher laser-plasma coupling. NCD targets which can be compatible to high repetition lasers may advance the acceleration mechanism to experience the high-energy ions at high repetition rate. Recently investigations where MVA mechanism has been studied theoretically^22–25^ and experimentally^27,28^ and concerned a excessive agreement owing to its estimation for attaining relativistic electron and proton beams.

Gas jet targets bid the opportunity for high repetition rates^29,30^, but so far they have been limited to low particle energies and yields^27^. Recently^31^ liquid targets have also shown a number of attractive features for meeting real world needs and can be rapidly delivered into the interaction region, and mitigate debris. In our former simulation research^25^, proton acceleration with tightly focused linearly polarised (CP) PW laser pulses (2 PW-20 fs) was realised in NCD plasmas (~1.0n_c_). The simulation consequences were obtained in a preionized near critical and above critical density hydrogen plasma which is still challenging to accomplish practically.

In this article, we demonstrate for the first time the generation of self-modulated high energy proton micro-bunches, of high energy and monochromaticity, driven by high-intensity short laser pulses from a novel plasma target of mixed ion species^34^, specifically a gas target consisting of C and H atoms. Such plasma states can occur from the effectively instantaneous molecular dissociation and above barrier ionization^35^ of CH4 or other simple alkane gases, due to the high intensity of the prepulse for PW-class lasers. Hence, in our simulations, we consider a pre-ionized plasma (consisting of C and H fully ionized nuclei). Due to transverse (radial) field components of the plasma field in the generated plasma channel, micro-bunching of proton beams has been observed. These microbunches are naturally spaced at the plasma wavelength which may also favour the generation of a strong plasma wake. These observations could also contribute significantly to the advanced proton-driven plasma wakefield acceleration research (AWAKE project^36^) which has recently demonstrated proton-driven plasma wakefields.

Our simulation results illustrate the generation of high-quality proton micro-bunches, of vital relevance for various applications such as proton cancer therapy, via a new laser-driven acceleration regime, which utilizes few-cycle (20 fs) high power (2 PW) laser pulses and gaseous targets. Such laser pulses are expected to become available, in the near future, at upcoming large scale facilities, such as the ELI project^37^, while the use of gaseous targets holds great potential for high-repetition rate operation of the laser-driven accelerator, free from plasma debris which can disrupt the laser focusing optics and from EMP generation which can disrupt major optomechanical components, such diagnostic electronics and motorized stages for target handling. It has also been seen that the interactions between two or more ion species during plasma expansion can significantly modify the final ion spectral distribution^17,34^. The specific changes to the spectra depend on the ions spatial distribution within the plasma and this provides an approach to achieving spectral control. The acceleration mechanism considered here seems to be a combination of ponderomotive acceleration, shock acceleration^38,39^, and multispecies expansion^40,41^. However, these mechanisms were not able to observe or show such microbunching or density modulation of proton beam. We emphasize in our simulation investigation of collimated proton microbunches of high energy which favours strong potential of utilising such mechanism for AWAKE kind of project.

## Laser-Plasma Interaction

**Figure 1:**
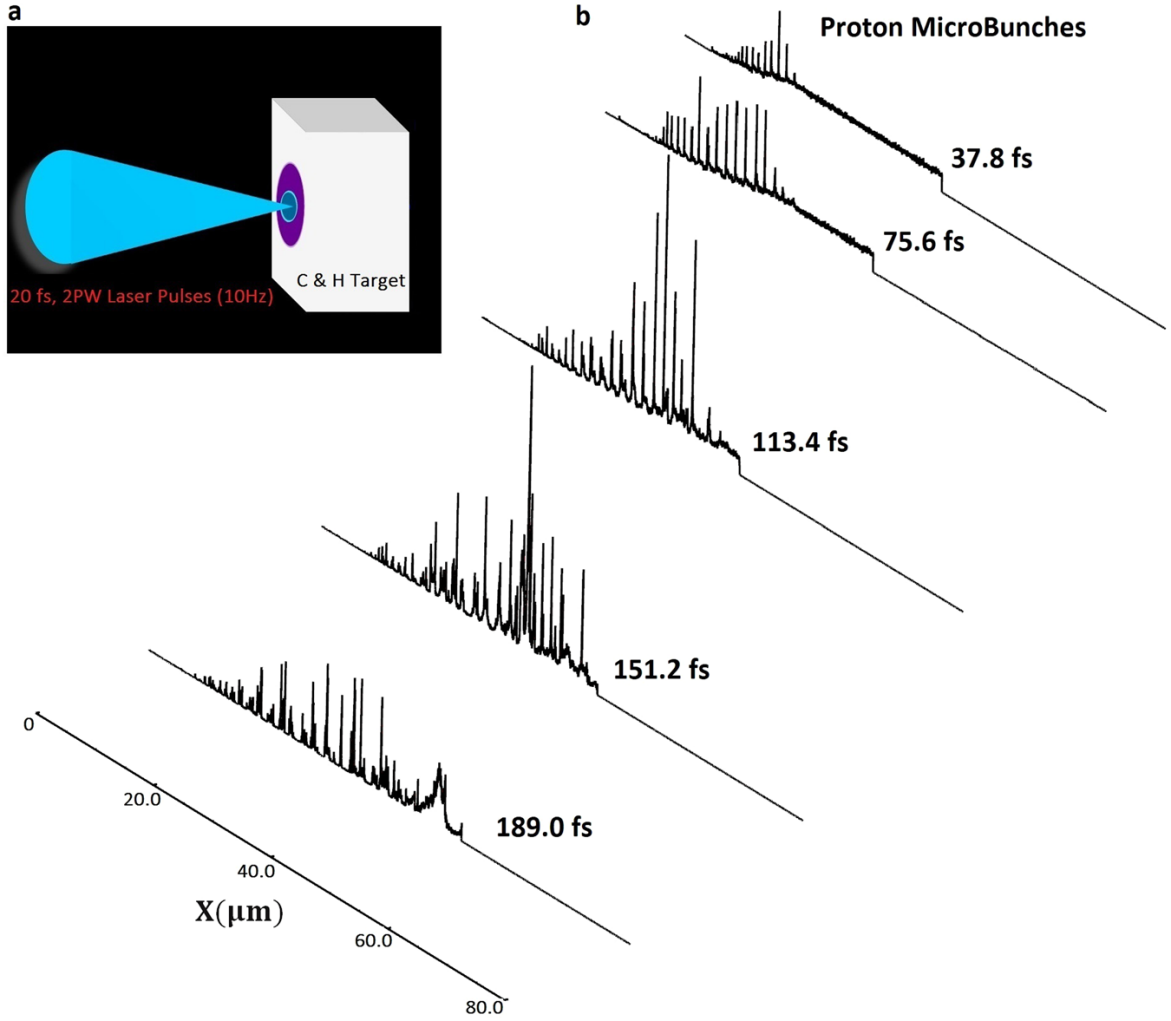
High energy proton micro bunches generated from the laser – plasma acceleration. (**a**) Schematic illustrations of laser-plasma setup for proposed ion acceleration mechanism from C & H target. (**b**) simulation output showing the generated proton micro bunches along the laser propagation direction (x-axis) at different time instants due to the interaction of an ultra-short, ultra-intense (2 PW, 20 fs) laser field with near-critical-density partially ionized plasmas (C & H species).

In this work, we numerically model the interaction of an ultra-short, ultra-intense (2 PW, 20 fs) laser field with near-critical-density partially ionized plasmas, via multidimensional PIC simulations using the PIConGPU code^42^. The laser pulse has 800 nm wavelength, a Gaussian intensity profile in space and time and is considered linearly polarized along the y-axis. The focused laser beam diameter on the C and H target is 5 μm (FWHM) and the laser pulse duration is 20 fs. The peak laser intensity is ~10^22^ W/cm^2^, and correspondingly, the normalized laser field is *a*_*L*_ = 68. Irradiation of a high-power laser pulse, the target is assumed to be partly pre-ionized due to realistic laser contrast and pre-pulses to C and H ions, while being slightly expanded on its surfaces (modelled as exponential density slope). The C and H plasma target is of slab shape with a thickness of 50 μm and is assumed to be non-magnetized, partially pre-ionized with the initial density of the fully ionized plasma to be 1n_c_, where n_c_ is the critical density for 800 nm light. The laser pulse is propagating along the x-axis and is focused onto the C and H target at normal incidence (as shown by Fig. 1). Figure 1 shows the generation of proton microbunches from C & H target at different time instants during the acceleration.

**Figure 2:**
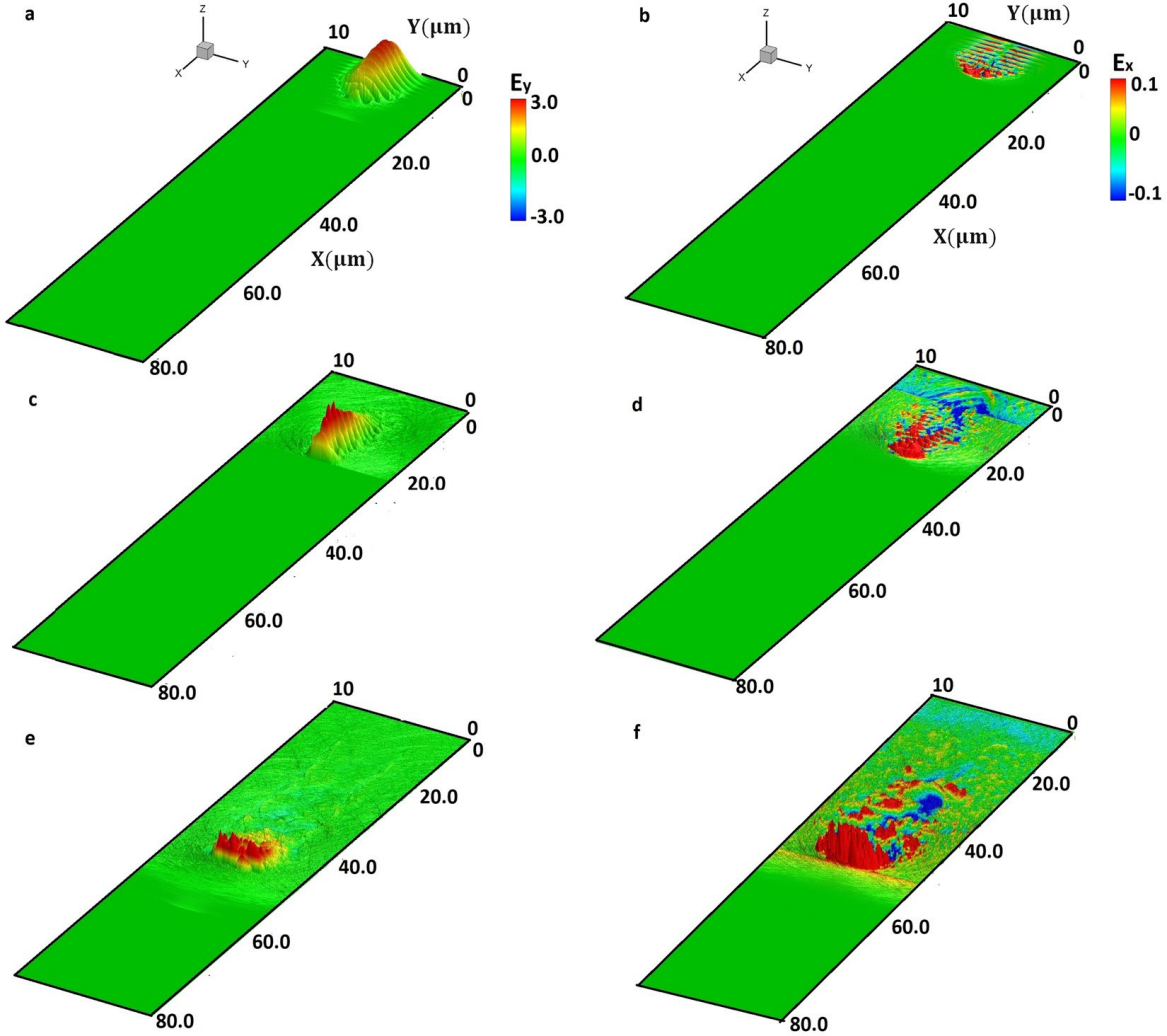
Spatio-temporal evolution of electric field of PW laser (E_y_) and plasma field (E_x_) at time instants 37.8 fs (**a**,**b**), 75.6 fs (**c**,**d**) and 151.2 fs (**e**,**f**) after the laser enters the target (*t* = 0). Sub-figures (a, c, e) show the spatio-temporal evolution of the laser field propagating in the C and H plasma and (**b,d,f**) the spatio-temporal evolution of the accelerating longitudinal field induced by the laser pulse in the generated plasma channel. The colour bar shows the variation of the laser field (in a, c, e) and the accelerating field (in b, d, and f) and is represented in TV/m.

In Fig. 2, the evolution of the laser field (a, c, e) and the longitudinal electric field (b, d, f) is illustrated at different time instants (*t* = 37.8 fs, 75.6 fs, 151.2 fs). Time instant *t* = 0, corresponds to time when the front part of the laser pulse hits the C and H plasma at its front surface in the XY plane. Initially, the intense (*a*_*L*_ = 68) laser pulse pushes drives the critical density surface (due to radiation pressure) forward at the hole boring velocity causing the creation of an ion density spike as it is shown later. Electrons (followed by protons) are pushed inwards by the radial ponderomotive force of the laser, along the laser propagation direction. After the initial laser hole boring stage, there is acceleration of plasma particles in the transverse and longitudinal directions. Subsequently, we observe the longitudinal field due to the shielded Coulomb repulsion where protons are accelerated by the electron-shielded carbon ions behind the protons. The transverse field in the plasma channel starts acting on the proton beam and modulates it. The modulation has a period very close to the plasma wavelength. Figure 2 demonstrates the wakefield structure generated behind the laser pulse which modulates the accelerated proton beam in microbunches.

## Mechanism of Ion Acceleration by Intense Laser Field

To better understand our observations, we start by describing the interaction of a linearly polarized laser with a partially pre-ionized plasma medium, such as our C and H mixed gas target. The irradiation of the front surface of the target causes the electrons in the skin depth from the front surface to be accelerated by the ponderomotive force

$${f}_{p}(r)=-\frac{{e}^{2}}{a.4{m}_{e}{\omega }^{2}}\nabla [{E}_{L}{(r)}^{2}(1-b.\mathrm{cos2}{\rm{\omega }}t)]$$

where *a* = *b* = 1 (linearly polarised case), and *a* = 2, *b* = 0 (circularly polarised case), *E*_*L*_ is the electric field of laser, *w* is the frequency of field oscillation, e is the electron charge and *m*_*e*_ is the electron mass and *t* is the time. The time-averaged component produces the steepening of the density profile, which applies a steady pressure to the target front surface and piles up the electrons into a compressed layer, inducing an intense charge separation field, which is responsible for the proton acceleration. The oscillating term causes heating and there is significant absorption of the laser field. The quasi-static electric field generated at the front surface of the target propagates with the hole boring (HB) velocity ${v}_{HB}/c={\varPi }^{1/2}/(1+{\varPi }^{1/2})=0.1c,$ where $\varPi =(Z/A)({n}_{c}/{n}_{e})({m}_{e}/{m}_{p}){a}_{L}$ and ${a}_{L}=e{E}_{L}/{m}_{e}\omega c$ here *n*_*c*_ is critical plasma density, *Z* is the charge state and *A* is the atomic number. The ions reflecting from the potential at HB surface acquire the velocity into the target as ${v}_{i}=2{v}_{HB}/(1+{v}_{HB}^{2}/{c}^{2})\approx 2{v}_{HB}$, which is known as hole boring. In near critical density targets the hole boring velocity is larger than the ion sound speed $({c}_{s}=\sqrt{Z{k}_{B}{T}_{e}/{m}_{i}})$, and it is equivalent to condition a_L_ > n_e_/n_c_.

We show in Fig. (2) the initial stage of laser interactions with near-critical density target where laser radiation pressure pushes electrons at the plasma surface, which sets up an electrostatic field originating from the charge separation that ultimately accelerates protons and carbon ions in the forward direction. Initially, the heavier carbon ions are left behind the lighter protons and electrons, and a triple layer system of propagating ions, protons and electrons is formed. The laser pulse proceeds to push the plasma surface, which has the critical density target, into the C and H target which is known as laser hole boring.

## Plasma Valley of Electrons, Protons and Carbon Ions

**Figure 3:**
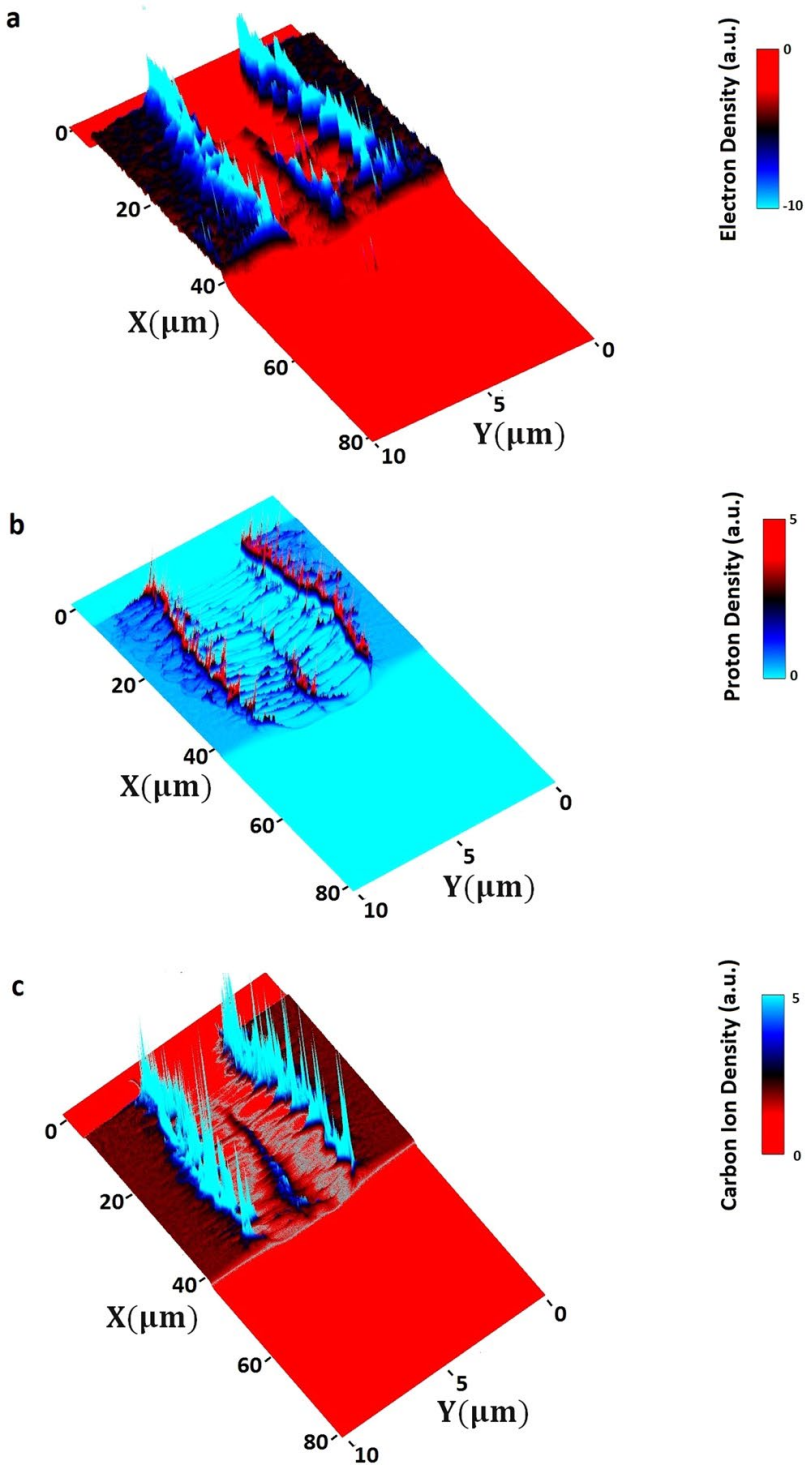
Illustration of the plasma valley (channel) formed by the PW laser pulse. Density distribution of (**a**) electrons, (**b**) protons and, (**c**) carbon ions are shown at time instant 151.2 fs when laser pulse exits from the rear side of plasma.

**Figure 4:**
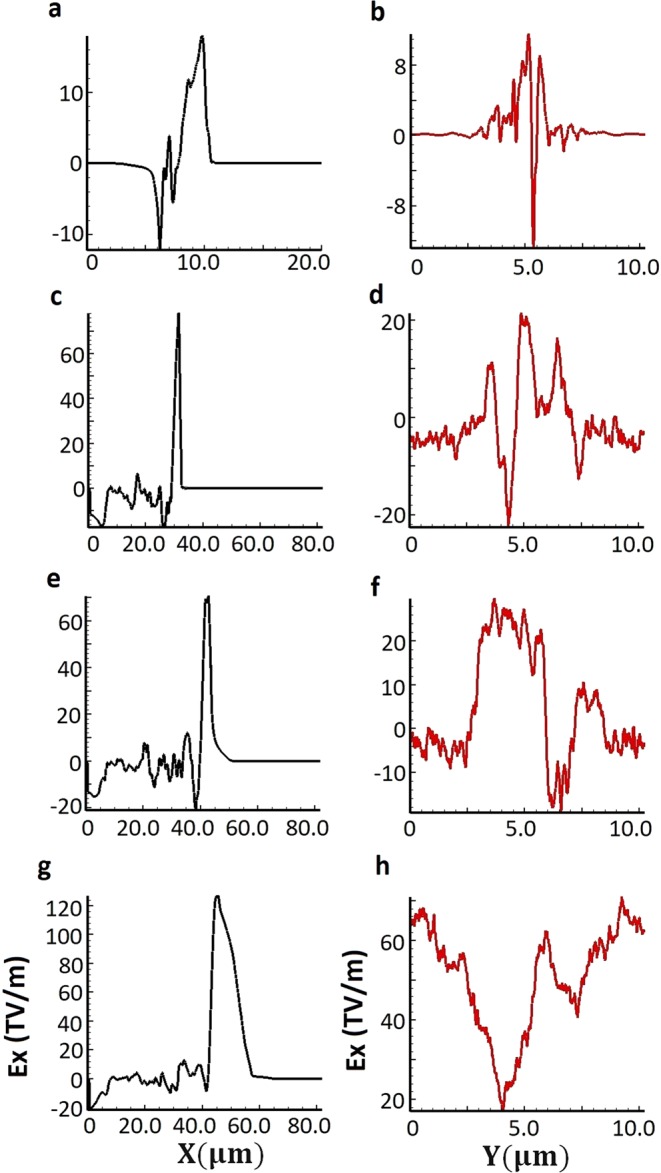
Evolution of accelerating field along the longitudinal and transverse direction of propagation. Snapshots in left panel (**a**,**c**,**e**,**g**) shows the variation of accelerating field (E_x_) along the longitudinal direction of propagation (ie. along the direction of laser propagation) and plots in right panel (**b**,**d**,**f**,**h**) illustrates the variation of accelerating field (E_x_) in transverse (radial) direction. Plots (**a**,**b**), (**c**,**d**), (**e**,**f**) and (**g**,**h**) corresponds to time instant of 37.8 fs, 113.4 fs, 151.2 fs and 189.0 fs respectively.

We show in Fig. 3, the density distribution of the electrons (a), protons (b) and carbon ions (c) at the time instant when the depleted laser pulse transmits from the rear side of the C and H plasma target. By this time, the laser pulse has been passed through the NCD plasma target and has formed a valley (channel) from which the electrons, protons and ions are expelled in the transverse direction to the laser propagation direction. Besides the longitudinal accelerating field, we also illustrated in this investigation the key role of the transversal variation of the accelerating electrostatic field as shown by Fig. (4).

## Spatio-temporal Evolution of Accelerating Field and High Energy Acceleration of Self-Modulated Proton Micro Bunches

The primary interaction of electrons in near critical density plasma with the electromagnetic field of laser pulse allows the acceleration of electrons and gain in electron energy. As an intense laser pulse propagates in near critical density plasma which provide favourable conditions for electron acceleration well beyond the ponderomotive potential, as laser pulse propagates with phase velocity (*v*_ph_) close to the speed of light. With a higher intensity laser pulse, electrons are expelled from regions of highest intensity and, if the ponderomotive force persists to balance the electric field acting to return the electrons, a cavitated channel can form which has two discontinuous sharp boundaries. In this regime, direct laser acceleration^39^ (DLA) assisted by quasi-static transverse (as shown by Fig. 4 right panel) and longitudinal (as shown by Fig. 4 left panel) electric fields of the channel may become the dominant mechanism generating an electron population with characteristic energies many times greater than ponderomotive energy.

Here, the two-dimensional particle-in-cell simulations show one of the dominant energy transfer mechanisms into the high-energy tail is mediated by the evolving longitudinal electric fields within the main plasma volume favouring the ions to experience huge, rapid acceleration via this mechanism. This is in stark contrast to previously identified DLA mechanisms^43^ that have either occurred in the very underdense region or essentially in vacuum with the overdense region serving as a source of electrons.

## Phase Space Distribution of Protons

**Figure 5:**
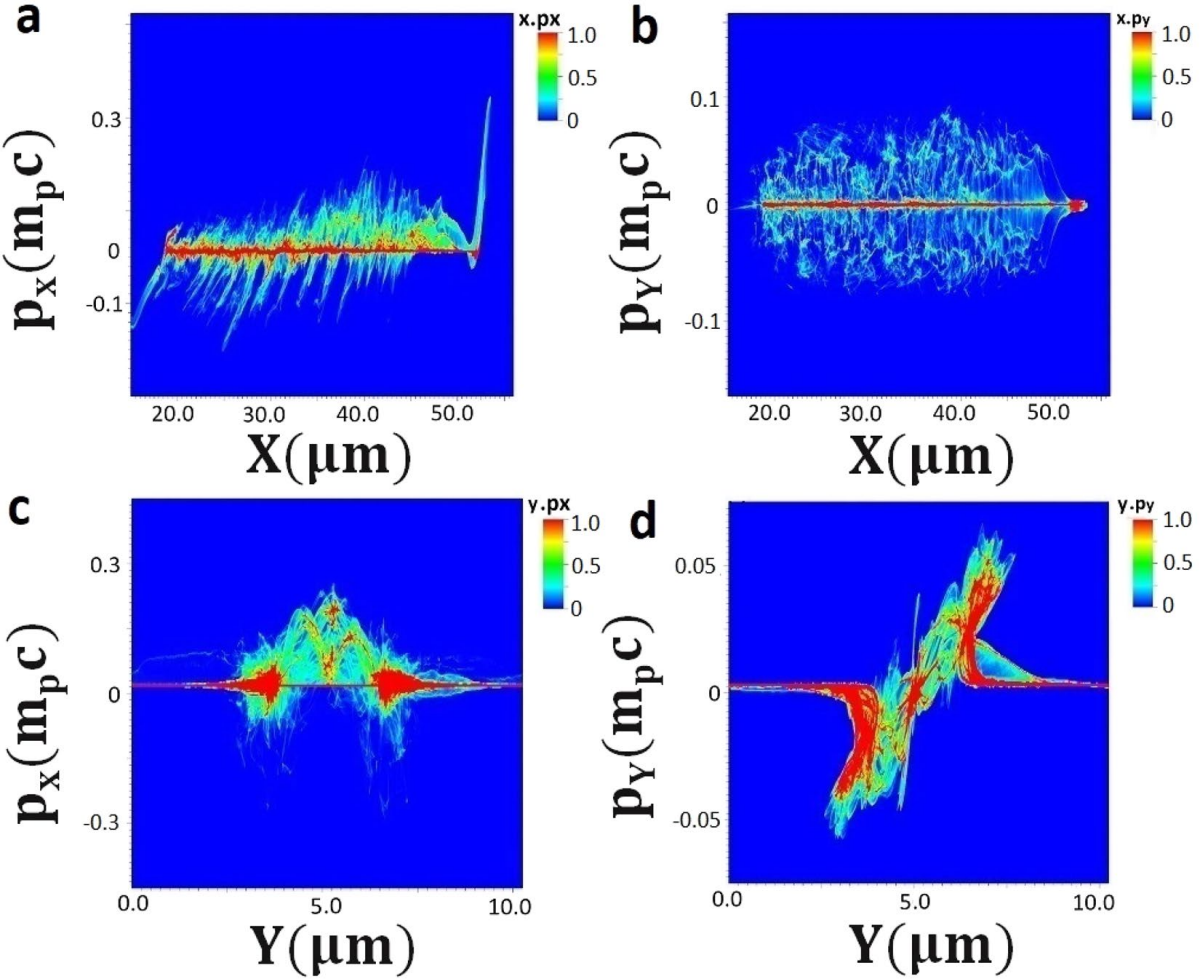
Illustration of ion phase space distribution at time instant 151.2 fs. (**a**,**c**): longitudinal momentum distribution (*p*_*x*_) and; (**b**,**d**): transverse (radial) momentum distribution (*p*_*y*_) of protons along the laser propagation direction (*x*-axis) and transverse to it along the *y*-axis. Color bar shows the variation of product of spatial dimension and momentum component and expressed in arbitrary units. Proton momentum is normalised with the m_p_c.

Figure 5(a–d) shows the phase space distribution of protons where longitudinal (*p*_*x*_) and transverse momentum (*p*_*y*_) is depicted along the direction of laser beam propagation and in transverse (radial) direction. The large populations of protons are seen to be accelerated along the propagation direction. In Fig. 5(a), several shock structures can be seen which confirms the generation of proton micro bunches and their acceleration to higher energies due to the dominant longitudinal field over the transverse plasma field (as depicted in Fig. 4). The transverse (radial) momentum of ions along the propagation direction shows the radial acceleration of ions as well as shown in Fig. 5(b). Figure 5(c) shows the longitudinal momentum distribution (p_x_) in y-direction (transversal to longitudinal direction of propagation). The central axis of propagation lies around y = 5 micron and the boundaries of plasma channel formed by the laser lies around 2.5 micron (y < 5 micron) and 7.5 micron (y > 5 micron). Near the boundaries of plasma channel there are ions accelerated in forward and backward direction. However large populations of ions, distributed along the central axis of propagation (y = 5 micron) which are mainly accelerated in forward direction as can be seen clearly now from Fig. 5(c). Figure 5(d) shows the transverse momentum distribution (p_y_) along the y-direction (transversal direction to the longitudinal direction of propagation). The variation of transverse momentum (p_y_) along the longitudinal direction (x-axis) shows that large populations of ions are accelerated along the central axis of propagation. Previous investigation^39^ has shown that at high density, plateau is seen in energy distribution of ions however results demonstrated here shows modulation in energy spectrum at near critical density due to periodic longitudinal field of shocks.

## Energy Distribution of Proton Beam

**Figure 6:**
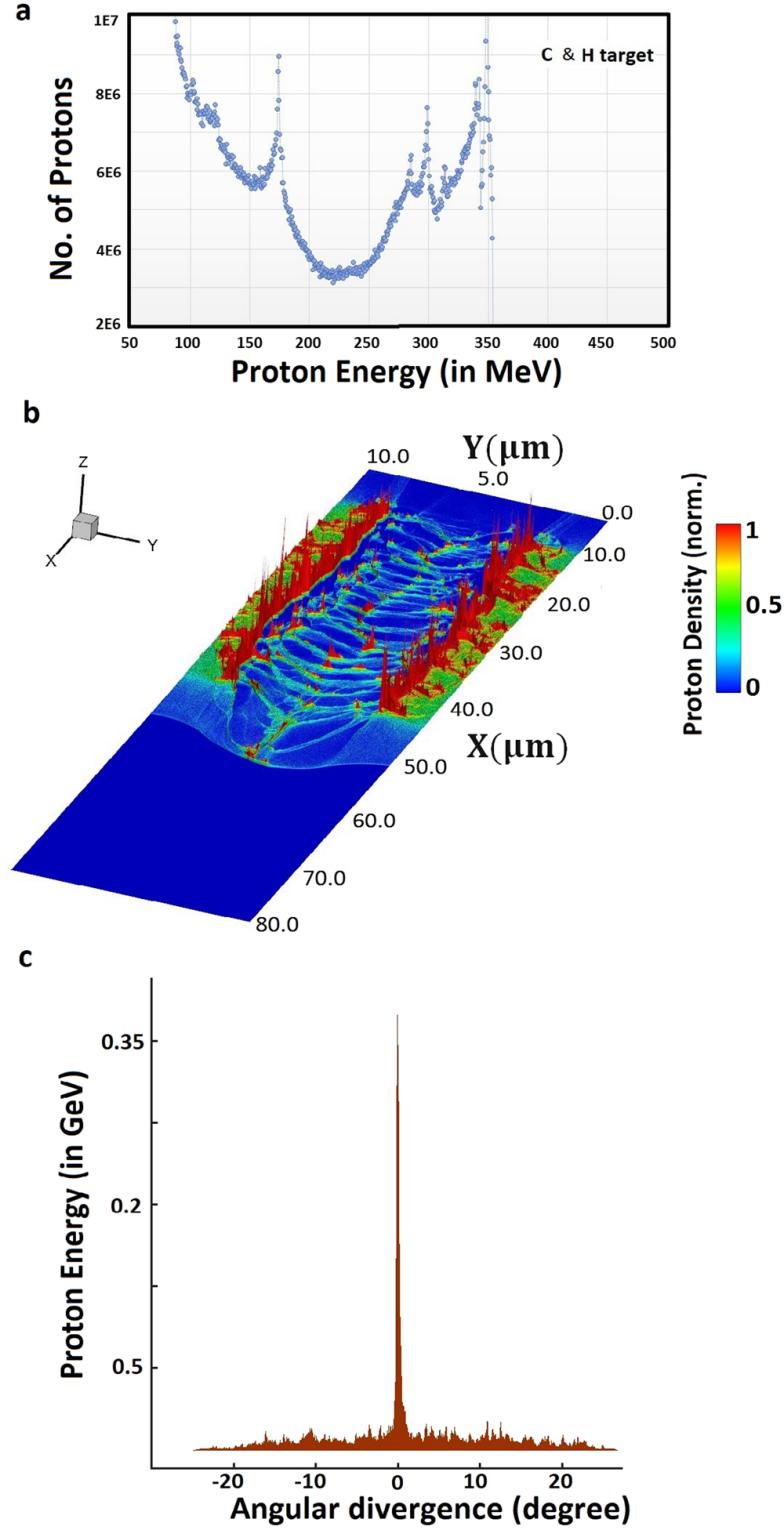
Portrayal of high energy – high quality proton microbunches. Proposed ion acceleration mechanism predicts here in shown simulation results (**a**) the energy distribution of proton beam with maximum proton energy 350 MeV, (**b**) density distribution of accelerated and self-modulated proton microbunches, and (**c**) the angular divergence of proton beam at time instant 200 fs. The colour bar shows in (**b**) corresponds to the variation in energy density of proton bunches which is in arbitrary units.

We conclude here the ion beam quality obtained from the simulation results. We demonstrate in Fig. 6(a) the energy distribution of proton beam where we numerically observe the high energy protons at the rear side of target after plasma-vacuum interface. The energy spectrum of protons shows the maximum proton energy at 350 MeV. These high energy protons are accelerated in forward direction and large populations of these protons are distributed around the laser axis. Further, the proton beam is characterised (as shown in Fig. 6(b)) by showing the energy and spatial density distribution at the time instant of maximum acceleration. Figure 6(b) shows the spatial density distribution of the focused proton beam, where high-energy protons are concentrated in very small areas (micro-bunching). A spectral peak at 175 MeV has been shown in proton energy spectrum (as in Fig. 6(a)). To observe the directionality of proton beam we depicted in Fig. 6c the angular divergence of proton beam which shows that large populations of high energy protons are directed along the propagation axis of laser.

The efficiency of ion acceleration mechanism investigated in this report can be estimated from the energy transformation from laser to protons. The number of protons in the energy 100 MeV–350 MeV are more than 10^8^. The employed laser energy on target is 40 J. Thus, laser to proton energy transformation efficiency can be estimated as ~10^−4^.

**Figure 7:**
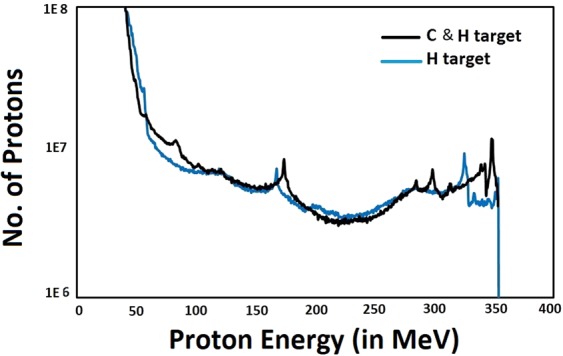
Comparison of ion energy distribution curves for target of mixed ion species (C & H) and pure H. Ion energy distribution curves are shown at time instant 200 fs (time when laser pulse propagates away from the target and ions are passed from rear side of plasma-vacuum interface).

Simulations conducted with the same laser parameters and plasma density profile, but with a pure hydrogen plasma, show that the spectrum of accelerated protons is similar to the case of a C and H plasma. This illustrates (as shown by Fig. 7) that the presence of multiple ion species does not significantly affect the maximum proton energies achieved, however the proton energy spectrum (with a C and H target) shows multiple peaks towards the higher energy tail which indicate the novel ion acceleration mechanism of high energy proton micro-bunches due to self-modulation of the proton beam in partially pre-ionized plasmas. This acceleration mechanism is of high relevance to the AWAKE experiment^36^, where a proton beam propagating in plasma self modulates into a series of microbunches. In the AWAKE experiment the proton bunch and laser propagate in a long rubidium (Rb) vapour where the laser pulse singly ionises the Rb vapour to form a plasma. The ionised Rb plasma then interacts with the proton beam and modulates the long proton beam into a series of microbunches which drives a strong wakefield in the plasma.

## Outlook

The present work is the first simulation demonstration, for the generation of energetic, laser-driven proton micro-bunches, while monitoring the acceleration and self-modulation of the proton beam. The new mechanism employs intense and short laser pulses interacting with gas targets of mixed ion species (C & H). Through this acceleration mechanism we observed high energy monochromatic proton microbunches of high quality (peak proton energy 350 MeV, laser to proton conversion efficiency ~10^−4^ and angular divergence <10 degree), which can be of high importance for medical applications. The simulation findings open up a new direction to the advanced proton-driven plasma wakefield acceleration research (AWAKE project) which has recently demonstrated proton-driven plasma wakefield by self-modulated proton microbunches. Understanding of such particle acceleration mechanism is of high impact to the development of laser-driven ion acceleration, given that many of the promising schemes employ near solid density target.

This computational study outlines the laser-plasma parameters necessary for the experimental demonstration of such high-repetition rate, laser-driven proton sources, free of plasma debris and EMP disruptions due to the gaseous nature of the target. The proposed plasma density window is preferred in order to get experimental access of near critical density gas targets^25–31^, which allows for the high flux of ions by employment of high-repetition rate, PW-class laser systems^44^. Currently, few PW-class laser systems^44^ are already in operation and advanced laser facilities such as, Extreme Light Infrastructure, will be able to provide laser pulses with several PW at the unprecedented operational repetition rate of 10 Hz. The choice of laser-plasma parameters in this proposed scheme is based on the upcoming, ultra-short, high repetition rate, PW laser systems, such as the HF-PW laser system to be installed at ELI-ALPS^37^.

Thus, the results are not only of fundamental importance to understand the ion acceleration mechanism and generation of high energy proton microbunches from near solid density dual species gas target but it also involves the ultrashort – ultraintense laser pulses, which could profoundly affect the development of laser-driven particle sources.

## Methods

### Particle-in-cell simulations

In this report we performed simulations using the relativistic 2D3V PIC code, PIConGPU^42^, to illustrate the mechanism of ion acceleration and explore further the new acceleration mechanism involved in it. In the proposed simulation linearly polarized PW laser pulses (800 nm wavelength laser) propagates along the x-direction through the simulation box. The transverse and spatial dimensions of simulation box is 10 × 80 µm^2^. The laser pulse incidents on target from the left side of simulation box. We consider here the spatial-temporal profile of laser beam as a Gaussian distribution. The laser beam of pulse duration 20 fs (FWHM), and it is focused to a bean size of 5 µm (FWHM). ${a}_{L}$ = 68 (corresponding to focused laser intensity) is the normalised laser field amplitude. The simulations were performed for longer time duration of 300 fs to ensure the complete dynamics of laser pulse through the target.

CH target considered in each simulation run is representing C and H plasma of density 1.0*n*_*c*_ (critical density *n*_*c*_ = 1.74 × 10^21^ cm^−3^). In longitudinal and transverse direction, we considered 100 cells per wavelength. The time step is 19.0 asec in this 2D3V simulations. For each species we considered the eight computational particles per cell.
